# Why Flavins Are not Competitors of Chlorophyll in the Evolution of Biological Converters of Solar Energy

**DOI:** 10.3390/ijms14010575

**Published:** 2012-12-27

**Authors:** Mikhail S. Kritsky, Taisiya A. Telegina, Yulia L. Vechtomova, Andrey A. Buglak

**Affiliations:** A.N. Bach Institute of Biochemistry, Russian Academy of Sciences, House 33, Building 2, Leninsky Prospekt, Moscow 119071, Russia; E-Mails: telegina@inbi.ras.ru (T.A.T.); vechtomova@inbi.ras.ru (Y.L.V.); andreybuglak@gmail.com (A.A.B.)

**Keywords:** Evolution, flavin, flavoprotein photoreceptors, DNA photolyase, photosynthesis, model of prebiotic processes, photophosphorylation, ATP, light-harvesting antenna

## Abstract

Excited flavin molecules can photocatalyze reactions, leading to the accumulation of free energy in the products, and the data accumulated through biochemical experiments and by modeling prebiological processes suggest that flavins were available in the earliest stages of evolution. Furthermore, model experiments have shown that abiogenic flavin conjugated with a polyamino acid matrix, a pigment that photocatalyzes the phosphorylation of ADP to form ATP, could have been present in the prebiotic environment. Indeed, excited flavin molecules play key roles in many photoenzymes and regulatory photoreceptors, and the substantial structural differences between photoreceptor families indicate that evolution has repeatedly used flavins as chromophores for photoreceptor proteins. Some of these photoreceptors are equipped with a light-harvesting antenna, which transfers excitation energy to chemically reactive flavins in the reaction center. The sum of the available data suggests that evolution could have led to the formation of a flavin-based biological converter to convert light energy into energy in the form of ATP.

## 1. Introduction

Since the Precambrian Era, the conversion of energy from solar light in the biosphere has been performed almost exclusively through chlorophyll-based photosynthesis [1], and there are reasons to believe that “…photosynthesis began early in Earth’s history but was probably not one of the earliest metabolisms, and … the earliest forms of photosynthesis were anoxygenic, with oxygenic forms arising significantly later” [2]. The core event in the photosynthetic conversion of energy is the photoexcitation of chlorophyll in the reaction center (RC), which is followed by the involvement of the excited pigment in electron transfer. The electron transport chain (ETC) associated with a lipid membrane produces a proton-motive force, pumping H^+^ ions across the membrane. The electrochemical proton gradient then drives ATP synthase to catalyze the attachment of free orthophosphate residues onto adenosine diphosphate (ADP) to form an energy-rich phosphoanhydride bond in the ATP molecule. Alternatively, the electron transfer can lead to the conservation of energy in a strong biological reductant—a reduced molecule of the nicotinamide coenzyme. Operating both photosystems (PS1 and PS2), the chloroplasts of plants transform the energy from absorbed photons into the energy of ATP molecules and reduced nicotinamide coenzyme (NADP-H) *i.e.*, the molecules that provide energy and reducing power for CO_2_ assimilation. However, under certain conditions, ATP may be the only product from the photosynthetic conversion of light energy. This is the case during cyclic photophosphorylation in plant chloroplasts in which electron flow begins in PS1, is transferred via ETC and then returns to the PS1 chlorophyll. In photosynthetic purple bacteria, a single photosystem is involved in cyclic photophosphorylation and only produces ATP.

The RC chlorophyll does not necessarily need to absorb a photon to achieve an excited state, as its excitation can be the result of Förster resonance energy transfer from excited antenna pigments. The antenna expands the cross-section of the light energy-absorbing unit and the effective spectral range of acting light. All the major members of the system, *i.e.*, the photon-absorbing pigments, ETC and the ATP-producing mechanism, are structurally and functionally associated with the lipid membrane [3].

We know that evolution can create alternative versions of this light-energy converter. One example is a mechanism discovered in cells of the halophilic archaeon *Halobacterium salinarum* (*H. halobium*). This mechanism is based on the activity of bacteriorhodopsin, a retinal-binding protein that is an integral protein in the membrane of this organism and absorbs green light with a maximum wavelength of 568 nm. The excitation of the retinal molecule after it absorbs a photon changes the conformation of the protein and evokes proton pumping. When acting as a light-driven proton pump, bacteriorhodopsin transports protons from the inside of the cell to the outside of the cell to form an electrochemical proton gradient across the membrane. Similar to the mechanism of photosynthetic phosphorylation, the proton gradient drives membrane-bound ATP synthase to catalyze the formation of ATP [4]. Thus, as in the case of photosynthesis, both the photoprocess and ATP synthesis are associated with the lipid membrane. However, when compared to photosynthesis, this mechanism is a truncated version of a light energy converter: Intermolecular electron transfer does not mediate the formation of the proton gradient that occurs after the excitation of bacteriorhodopsin. Additionally, the excitation of bacteriorhodopsin does not involve the accessory antenna pigments.

This review discusses the existence of another potential version of the light energy converter, the activity of which is based on the excitation of flavins.

The main facts that form the basis of this hypothesis are as follows.

(1) Flavins are evolutionarily ancient molecules; (2) The flavin photocycle can lead to the accumulation of free energy in the products, and this cycle (in the chemical model) can provide the formation of high-energy phosphate: ATP; (3) Evolution repeatedly selected flavins to function as flavoprotein photoreceptors, and, as a result, organisms today utilize several families of flavoprotein photoenzymes and sensory photoreceptors. It is remarkable that, during evolution, one of these families has acquired a light-harvesting mechanism that is similar to the antenna of the photosynthetic apparatus and increases the flow of photon energy to the reaction (photochemical) center.

## 2. Flavins Are Evolutionarily Ancient Molecules

**Figure 1:**
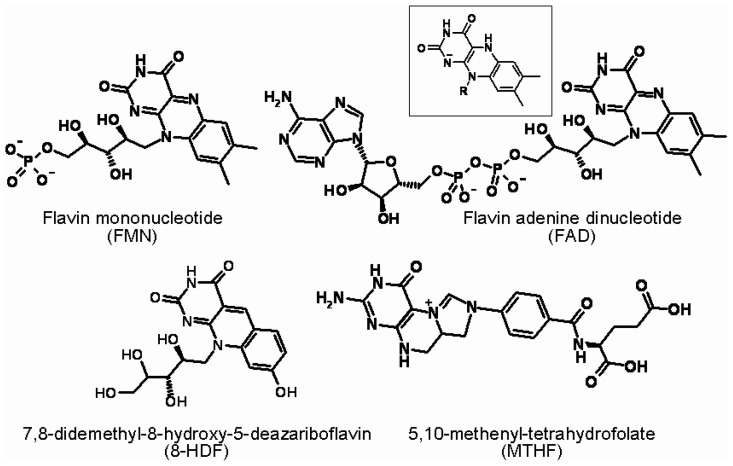
Some coenzymes of “dark metabolism” act as chromophores in photoreceptor proteins. Flavin molecules, riboflavin-5′-phosphate (also known as flavin mononucleotide (FMN)) or flavin adenine dinucleotide (FAD) (anionic dihydroflavin H-FAD^−^_red_ in DNA photolyases) function in the photocatalytic (reaction) center. Coenzyme molecules also act as photon harvesters in some flavoprotein photoreceptors, including 5,10-methenyl-tetrahydrofolate (MTHF) or 8-hydroxy-7,8-didemehyl-5-deazariboflavin (8-HDF), which is present in different organisms. FMN and FAD also act as antennae for some organisms. The boxed formula represents the structure of reduced isoalloxazine, the basic ring of the flavin molecule in the anionic dihydro form of H-FAD^−^_red._

The derivatives of heterocyclic isoalloxazine (2,4-dioxo-benzo-[g]-pteridine), riboflavin-5′-phosphate (or flavin mononucleotide, FMN) and flavin adenine dinucleotide (FAD) are essential cofactors for a variety of “dark” biocatalytic redox processes (Figure 1). The fact that these derivatives are present in all living organisms and that all known types of cellular metabolism require these molecules to function suggests the evolutionary antiquity of flavins. Despite some differences in the metabolic reactions that form riboflavin from guanosine triphosphate in Archeae and Eubacteria, this biosynthetic pathway is considered to be evolutionarily ancient [5,6]. This pathway operates in plants, fungi and in many microorganisms, including Archeae; however, animals do not synthesize riboflavin but obtain it as vitamin B_2_ from their diet or from intestinal bacterial symbionts.

Model experiments have demonstrated that isoalloxazine derivatives could have been present in the prebiotic environment. For instance, the heating of an anhydrous mixture of certain amino acids to 150–200 °C resulted in the formation of flavins conjugated to amino acid polymers [7–9]. In an aqueous medium, these conjugates aggregate to form phase-separated structures, termed “Fox microspheres” [10]. The structural similarity of the flavin coenzyme molecules FMN and FAD to nucleotides suggests a role as photocatalysts and regulator molecules in the “RNA world”—a hypothetical version of the most ancient biota [11,12].

## 3. Flavin Molecules Display Photochemical Activity

The flavin molecules (Fl) can exist in three different redox states: oxidized, one-electron reduced (flavin free radical or semiquinone, HFl^•^) and two-electron reduced states (dihydroflavin, H_2_Fl_red_). The disproportionation of two semiquinone molecules gives rise to the formation of oxidized flavin and dihydroflavin [13,14]. In solution, *i.e.*, when not enzyme-bound, a mixture of oxidized and reduced flavin sets up an equilibrium in which a certain amount of semiquinone is formed. With free flavins at pH 7 only about 5% radical is stabilized in an equimolar mixture of oxidized and reduced flavin [14]. Depending on the pH of the surrounding medium, each of these different redox states can exist in three different protonation states [13]. Both a change in its redox state and the excitation of the flavin induces a significant (up to several orders of magnitude) shift in the molecule’s proton affinity. In addition to the change in electron affinity, this shift may contribute to the photochemical process of flavin photoreceptors [15].

The conjugated system of heterocyclic isoalloxazine of the oxidized flavin absorbs photons from the blue area and the UV-A and UV-B areas of the light spectrum. Neutral solutions of riboflavin, FMN and FAD have a long-wave absorption maximum of 450 nm, and their absorption bands in the UV area have maxima at 375 nm and 260 nm. The molar absorption coefficient for the long-wavelength absorption maximum of flavin is relatively high (for example, FMN) at 450 nm it is 12.1 mM^−1^·cm^−1^. Blue light photons energize flavin with 2.75 eV (266 kJ·mol^−1^), and the excited molecule becomes highly electrophilic and can accept electrons from the donor. The reaction occurs in the presence of a wide range of substrates including amino acids, carboxylic acids, thiols, aldehydes, amines and unsaturated hydrocarbon [13,16,17]. The acceptance of a one-electron equivalent (either a hydrogen atom, or an electron) from the donor yields a semiquinone, *i.e.*, the flavin free radical (HFl^•^) [13,18–20]. The flavin semiquinone plays a key role in the photocycle both in solution and in the flavin-binding photoreceptor proteins. The addition of another electron to this free radical transforms it to the two-electron reduced species, dihydroflavin (H_2_Fl_red_). In the process of photoreduction the excited flavin can also bind to the donor’s residue (R) to form an adduct, R-FI_red_H [13,21]. The photoreduction of the flavin molecule becomes cyclic with the perpetual regeneration of the flavin pigment when the photoreduced flavin is reoxidized or the adduct is split.

The absorption bands of dihydroflavins are shifted in the UV region and they absorb light much weaker than the oxidized molecules. The neutral dihydro-FMN has the long-wavelength absorption maximum at 390 nm (ɛ = 2.4 × 10^−3^ M·cm^−1^) and the maximum for its anionic form is situated at 350 nm (ɛ = 5.4 × 10^−3^ M·cm^−1^) [22]. The excitation of the dihydroflavin molecule in the presence of an electron acceptor can also yield the flavin free radical; this reaction is an important step in the photocycle of some flavoprotein photoreceptors [23].

The redox processes involving excited flavin are influenced by the concentration of dioxygen in the medium: Photoexcited flavin generates singlet oxygen in the presence of dioxygen. The quantum yield of singlet oxygen for riboflavin, FMN and FAD is 0.5/0.54, 0.5/0.51 [24,25] and 0.07 [25], respectively. In an anoxygenic medium, the photochemical process is based entirely on direct electron exchange of excited flavins with a donor. In the presence of oxygen, an excited flavin can be involved in two processes, and oxygen enrichment decreases the proportion of molecules participating in a direct electron exchange at the expense of molecules transmitting energy to dioxygen to excite it to the singlet state [26]. Both singlet oxygen and flavin are strong oxidants such that both processes generate the same product—an oxidized donor.

## 4. Flavin Can Convert Light Energy into the Energy of ATP: A Prebiotic Process Model

The products of flavin-photocatalyzed reactions accumulate free energy; for example, flavin molecules can convert light energy into chemical energy. Excited flavins oxidize donors with a more positive electrode potential than that of the Fl/HFl^•^ pair (*E*_o_′ = −0.31 V) [27]. Researchers often use ethylenediaminetetraacetic acid (EDTA) as a donor with a relatively positive potential [28–30]. The *E*_o_′ value for EDTA is equal to +0.40 V) [31]. In the presence of EDTA, excited coenzyme flavin molecules and abiogenic isoalloxazine pigments transit to the reduced state, or (in the presence of an electron acceptor, for instance, Fe^3+^-cytochrome *c*) they photocatalyze the reduction of this acceptor [32,33].

To date, there is no evidence for flavin-sensitized ATP production in organisms. However, this process occurs in a model that mimics the prebiotic environment [34,35]. This model was based on the activity of abiogenic template-bound flavin, which exhibits photochemical activity; the template refers to the host polymer substance within the photochemical context. This polymer reversibly binds and concentrates the substrate and creates catalytic centers yet does not become part of the product [36]. The organic components of the photophosphorylating system, polyamino acid matrix (the template) and flavin chromophore are the products of the thermolysis of anhydrous mixtures of amino acids (glutamic acid, lysine and glycine) in an anoxygenic medium [7–9]. When this product contacts water in the presence of orthosilicic acid, it self-aggregates to form the so-called “Fox’s proteinoid microspheres” [10,33]. Irradiation of this microsphere suspension with blue or UV-A/B light causes the phosphorylation of ADP by orthophosphate to form ATP. Under these conditions, the molar yield of ATP exceeded 20%, and the quantum efficiency was approximately 0.15/0.20.

The action spectrum of the photoprocess indicated that flavin was involved in photophosphorylation, and the process exhibited maximum activity when the suspension of microspheres was irradiated by light with wavelengths of 450 and 375 nm. These maxima are very close to the absorption maxima of flavins. A change in the composition of the precursor amino acid mixture, the replacement of glycine with alanine, changed the product of thermolysis to pteridine instead of the flavin pigment, and the abiogenic pteridine photocatalyzed the phosphorylation of ADP to form ATP. The spectral efficiency of the process moved to the UV-A area, which is typical for the absorption of pteridines, with a maximal activity at about 350 nm, and the product yield was reduced [37].

However, the mechanism of phosphorylation in this model is unclear, and attempts to identify analogs in other organisms have not been successful. The photophosphorylation process was sensitive to superoxide dismutase, and activity depended on the presence of agents that could ensure the oxidative regeneration of flavin, including dioxygen, hydrogen peroxide, or “non-oxygenic” electron acceptors (e.g., Fe^3+^-cytochrome *c* (*E*_o_′ = +0.25 V)) [33]. Thus, photophosphorylation is related to light-induced electron transfer and likely involves the formation of a flavin-free radical. According to one hypothesis, the mechanism involves an interaction between this flavin-free radical and ADP to form an ADP-free radical, which is then subjected to phosphorylation. Such a mechanism has been proposed for the riboflavin-sensitized phosphorylation of ADP in solution [38,39]. Because the yield of ATP in previous studies was small, not exceeding 0.1%, the effectiveness of the flavoproteinoid model can be attributed to the influence of the polyamino acid-silicate template. When discussing the reaction mechanism, we should consider a possible role of the silica matrix in facilitating the catalytic transfer of phosphate ions. Recently, the effect of silicate minerals on the phosphorylation of sugar molecules in a mimicked prebiotic environment has been reported [40]. Moreover, ultraviolet irradiation of the ADP molecules, which are adsorbed to the particles of the clay mineral montmorillonite, leads to their phosphorylation by orthophosphate and the formation of ATP [35].

## 5. Biological Evolution Repeatedly Chose Flavins for Light Receptors

In the 1950s, Krasnovskii suggested that the up-hill reactions photocatalyzed by nicotinamide and isoalloxazine coenzymes or their abiogenic analogs, could have been a prototype for photosynthesis [41]. Since then, studies have shown that prebiotic environments could achieve the abiogenic synthesis of flavins and the subsequent formation of flavin-based converters of light energy. However, the question remains of how reasonable it is that flavin converters could exist in biological systems.

Biological evolution did not reject flavin photochemistry, and these molecules are involved in the physiological reception of light. Recently, several families of photoreceptor flavoproteins have been discovered in organisms from all kingdoms. These photoreceptors are not involved in the energy supply of organisms, but they do participate in organismal adaptation to the environment.

For example, the photoenzyme DNA photolyase repairs molecular lesions in DNA following UV-B irradiation. The repair is a photochemical process in which the irradiation-induced cross-links between two adjacent pyrimidines are split by excited flavin in the catalytic center of the photolyase enzyme [42,43]. Structurally (but not functionally) similar flavoproteins, known as cryptochromes, function as receptors of blue and UV-A light in the photoregulation of metabolism and development [44,45]. The participants of the photoregulatory processes are also proteins, which bind the flavin chromophore via its LOV or BLUF domain [46,47]. In biochemical terms, these photoreceptors are input units of the light-activated cascades of cell regulation. Some of these photoreceptors participate in the control of gene expression [48,49], and other photoreceptors are involved in the control of cyclic nucleotide concentrations or are members of protein kinase cascades, the activity of which depends on the excitation of the flavin chromophore [50–52]. The structural differences between flavoprotein photoreceptor families indicate the absence of a common ancestor sequence. In other words, throughout the history of the biosphere, the evolutionary process has repeatedly turned to flavins as suitable participants in light reception [53].

The photochemically active flavin chromophore in cryptochromes and DNA photolyases is FAD; in the latter protein, it is present as the anionic dihydro form, H-FAD^−^_red_ [43,44]. The BLUF domain also binds FAD, and the LOV domains of some photoreceptors (the fungal ones) bind FAD. Similar domains in plant proteins, phototropins, use FMN as the chromophore [35,54]. In most flavoprotein receptors, the photochemical cycle involves the excited singlet state of the flavin chromophore; the exception is the LOV domain in which the photocycle involves triplet flavin molecules [46,55–58], a fact that deserves attention within the context of evolution. Triplet flavin molecules have longer lifetimes than excited singlet states, allowing the triplets to participate in chemical reactions in unorganized media (e.g., in solution).

In contemporary organisms, the dominant position belongs to the photoprocesses involving short-lived singlet excited molecules. Examples include chlorophyll-mediated photosynthetic processes, and the activity of retinal-binding photoreceptor proteins such as bacteriorhodopsin or rhodopsin. These processes are functional only in spatially ordered media and require the participation of protein molecules and membrane structures.

## 6. How Do Excited Flavins Exhibit Chemical Activity in Photoreceptor Proteins?

The question arises of whether the photocycle of the flavin chromophore in a protein is similar to the cycle in simpler systems. The photocycles of flavin chromophores in the modern families of flavin photoreceptors differ markedly. In addition to the excited flavin, photochemical processes involve either the amino acid residues of the apoprotein or (in the case of DNA photolyase) the damaged region of the DNA molecule bound by the enzyme-substrate complex. In photolyase, the excited H-FAD^−^_red_ transfers an electron to a substrate, such as the pyrimidine dimer that is in need of repair, and the resulting free radical intermediate further splits into two pyrimidines. Once the repair is complete, the electron passes to the flavin, returning it to its original FADH^−^_red_ state. However, the photoinduced electron transfer process in cryptochromes differs from that in the structurally similar DNA photolyase proteins and is restricted by the protein molecule itself [44]. The electron source for the photocycle in cryptochromes, with the oxidized form of FAD being a photoreactive group, is not entirely clear. Presumably, tyrosyl and tryptophanyl residues of the apoprotein play this role. The presence of DNA-photolyase activity in some cryptochromes, which require dihydroflavin for the catalytic process, complicates this problem [59].

In the LOV domain of the plant blue light receptor phototropin, excited flavin interacts with the thiol group of one of the cysteinyl residues to form an adduct that splits in the dark to return the system to its original state [57,60,61]. The photoactivation mechanism of the Slr1694 BLUF domain from *Synechocystis* sp. PCC 6803 was shown to involve a proton-coupled electron transfer from a conserved tyrosyl residue to the photon-excited flavin chromophore that results in a neutral flavin semiquinone/tyrosyl radical pair formation (probably, *via* an intermediate formation of FAD^•−^ anion) [62–64]. Participation of the excited flavin in the electron transfer was shown also for the Blrb BLUF domain in the photoreceptor of *Rhodobacter sphaeroides* [55,65].

Thus, despite the difference in the details, the flavin photocycles in protein photoreceptors involve the participation of a chromophore molecule and electron transfer, with the formation of a free radical species.

## 7. Evolution Has Equipped Some Flavin Photoreceptors with Light-Harvesting Antennae

In evolutionary terms, it is interesting that some flavin photoreceptors have acquired antennae, increasing the efficiency of photon energy collection. Although the pigment composition and structural details of photosynthetic antennae vary in organisms belonging to various taxonomic groups [2], they do exhibit some common and fundamental similarities. In all organisms, photosynthetic complexes are oligomeric proteins, and the number of antenna pigments serving one RC exceeds one. This ratio ranges from tens of pigment molecules per RC in some Proteobacteria and Heliobacteria to several thousands of pigments per RC in the green sulfur bacteria. In oxygenic photosynthetic systems, *i.e.*, cyanobacteria and chloroplasts, one RC is supported by several hundred molecules of antenna pigments [66]. For example, a PS1 of cyanobacterium *Synechococcus elongatus* consists of 12 protein subunits and 127 cofactor molecules comprising 96 chlorophylls and 22 carotenoids [67].

Förster resonance energy transfer is the transfer of energy between a donor chromophore initially in its electronic excited state to an acceptor chromophore through nonradiative dipole-dipole coupling. As a result, the photosynthetic apparatus is able to consume both the energy of photons absorbed by the RC chlorophyll in the relatively narrow absorption bands of blue and red areas (for chlorophyll *a* λ_max_ = 410, 430 and 662 nm) and photons of a much broader range, essentially the entire visible spectrum (with some “subsidence” in the green area).

The transfer of energy between two dipoles occurs when these are suitably positioned and closely located in relation to each other, and the donor fluorescence emission spectrum overlaps the acceptor absorption spectrum. With respect to the photosynthetic mechanism, the photochemically active excited singlet (S1) state of an RC chlorophyll *a* (λ_max_ = 662 nm) can be supported by energy from the antenna pigments, which absorb photons of the entire visible spectrum. Evolution has selected a series of antenna pigments that absorb light at different wavelengths, and, chemically, these pigments belong to different classes, including chlorophylls (Mg-porphyrins), carotenoids (isoprenoids) and bilins (linear tetrapyrroles). For instance, the absorption maxima of the antenna’s chlorophyll *b* are 453 and 642 nm, and the β-carotene’s absorption maxima (in hexane) are 425, 451 and 480 nm. The algal pigment phycoerythrin has maxima at 490, 546 and 576 nm, and another algal bilin, phycocyanin, absorbs light with a maximum at 618 nm [68,69].

The absorptivity of antenna pigments matches or exceeds that of the RC chlorophyll. The molar absorption coefficients of photosynthetic pigments in their long-wavelength maxima, corresponding to the (S1) excited singlet state involved in energy and electron transfer, are given below. For RC chlorophyll *a* (λ_max_ = 662/663 nm), this value is 75.1/91.2 mM^−1^·cm^−1^ and the ɛ_665_ value of the antenna’s chlorophyll *b* is 47.0/51.5 mM^−1^·cm^−1^, depending on the solvent in both cases. The molar absorption coefficient of one of the antenna bilins, phycocyanobilin, in its long-wavelength maximum at 690 nm is 37.9 mM^−1^·cm^−1^ [68,69]. β-carotene is a powerful photon harvester, and the molar absorption coefficient of it in hexane solution at 451 nm maximum is 139.5 mM^−1^·cm^−1^ [70].

It is noteworthy that some flavoprotein photoreceptors, namely proteins of the DNA photolyase/cryptochrome family, are equipped with a light-harvesting antenna. Under relatively low light flux, this antenna increases the supply of excitation energy to the chemically active flavin chromophore. In the physiologically relevant blue and UV-A areas, the absorption coefficient of this flavin is low, approximately 2 mM^−1^·cm^−1^. To compensate for the low absorption, the excitation energy is transferred to FADH^−^_red_ from the second light-harvesting chromophore, which increases the activity of photocatalytic DNA repair proportionally with the rate of absorptivity of the two chromophores [43].

The DNA photolyase antenna is a heterocyclic molecule non-covalently bound to the apoprotein in such a way that its dipole can interact with the catalytically active flavin, molecules structurally similar to flavins and including pterin, 5,10-methenyl-tetrahydrofolate (MTHF) or the deazaflavin 8-hydroxy-7,8-didemehyl-5-deazariboflavin (8-HDF) in different organisms (Figure 1). As in the case of the chromophores in photocatalytic centers, the molecules acting as photon harvesters are essential cofactors of “dark” biocatalytic reactions. MTHF participates in monocarbon group transfers, and 8-HDF is a fragment of the F420 cofactor, which is involved in redox reactions in some prokaryotes [71,72]. The molar absorption coefficients in the absorption maxima of the antenna molecules are as follows: for MTHF, ɛ_365_ = 25.1 mM^−1^·cm^−1^, and for 8-HDF, ɛ_420_ = 38.5 M^−1^·cm^−1^. [73,74] These values indicate that MTHF and 8-HDF differ from structurally similar coenzymes and nucleotide molecules by a higher absorptivity in a physiologically relevant area of the spectrum (λ_max_ ≤ 320/330 nm) [53]. In some organisms, the oxidized forms of FMN and FAD (ɛ_450_ = 12.0 mM^−1^·cm^−1^) play this role, though with less efficiency than MTHF or 8-HDF [75,76]. Most recently, 6,7-dimethyl-8-ribityl-lumazine has been detected in the antenna-binding domain of a cryptochrome of *Rhodobacter spheroides* [77]; the absorption spectrum maximum for the 6,7-dimethyl-lumazine solution is 328 nm, with a molar absorption coefficient equal to 10.0 mM^−1^·cm^−1^.

The efficiency of photon absorption was not the only criterion for selecting the antenna pigments of flavoprotein photoreceptors. We can assume that the molecule’s resistance to photolysis and, in a broader sense, its low photochemical activity could favor the selection of flavoproteins. For example, MTHF is significantly more resistant to photodestruction than tetrahydrofolic acid and other reduced folates [78]. Paradoxically, the presence of oxygen increases the resistance of MTHF to light, which interestingly correlates with the fact that MTHF (as opposed to 8-HDF) plays the role of an antenna in organisms belonging to the strictly aerobic branch of the evolutionary tree.

The structural organization of the light harvesters for DNA photolyases and cryptochromes also differs from the photosynthetic antenna. Instead of numerous pigment molecules, each photocatalytic (reaction) center of the flavoprotein photoreceptor is serviced by a single light-harvesting chromophore molecule. In contrast to the photosynthetic apparatus, flavoprotein photoreceptors and their chromophores are water-soluble molecules and are not integral components of the lipid membrane. Unlike photosynthetic photosystems, which are multimeric intermolecular aggregates, DNA photolyases do not maintain oligomeric structures. The photocycles of some other flavoprotein photoreceptors involve the formation of homo- and heterodimers [47].

Thus, hypothetically, flavin energy converters may be supplemented by a light-harvesting antenna that increases the efficiency of the photon energy utilization. In structural terms, such a light harvester was invoked by different principles than the photosynthetic antenna.

## 8. The Environmental Framework for a Hypothetical Energy Converter

In discussing the possible role of flavins in the evolution of photobiological processes, we rely on the following data. Flavins were available in prebiotic processes. Flavin photocycles involve electron transfer, and (at least in the model) the photocycle can be coupled to ADP phosphorylation. Because flavins were repeatedly recruited to perform photoreceptor functions, we suggest that one such attempt could lead to the formation of a flavin-based converter of light energy. The data obtained in the study of this model allowed us to assume that such a converter did not require a transmembrane proton gradient to drive ATP synthesis (Table 1).

In addition to the bacteriorhodopsin mechanism, the hypothetical flavin-based converter can be regarded as a truncated version of the photosynthetic converter; one feature is that it can operate in the absence of a lipid membrane. However, the properties of flavins do not exclude their excited molecules from functioning within the membrane. For instance, an amphiphilic flavin derivative embedded into an artificial lipid membrane was shown to photocatalyze the translocation of redox equivalents across the membrane [79].

Among the selective advantages of the flavin-based converter, this converter was adjusted to the spectral profile of solar radiation reaching the early Earth. More precisely, the absorption spectrum of flavins nearly complements the emission spectrum of the F- and G-class stars. As for the Sun, it is a G2V star according to stellar classification [80] and emits maximum energy in the range of 475 nm to 505 nm [81]. The long-wavelength flavin absorption maximum at 450 nm is close to the maximum of solar radiation before the atmosphere was enriched with ozone. The emergence of an ozone layer has decreased the access of short-wave radiation (blue-violet visible light and UV-A and UV-B radiation), which is absorbed by flavins, and the radiation spectrum shifted toward the red region. The ratio of energy input reaching sea level at the borders of the visible spectrum in the UV and IR ranges (*E*_400_/*E*_750_) fell from approximately 1.5 to 0.9. We believe that this change in light conditions deprived flavins of an important selective advantage.

The question arises why we focus on the oxidized form of flavins as potential photoreceptor and ignore the two electron reduced or one electron reduced (semiquinone) molecules? This question is particularly relevant in the context of the modern concept that before oxygenic photosynthesis evolved, Earth’s atmosphere had practically no free oxygen [82]. And after all, modern photoreceptors use not only oxidized flavins, but also the anionic dihydro form, H-FAD^−^_red_, which acts in the catalytic center of DNA photolyases.

The dihydroform absorbs light significantly weaker than oxidized flavin and in a more narrow spectral range, which practically does not go beyond the ultraviolet region (see Section 3). To be efficient in photochemical processes, the H-FAD^−^_red_ molecule in the catalytic center of DNA-photolyase needs an inflow of energy from the antenna chromophore with higher absorptive capacity. For this reason, the functioning of fully reduced flavin as the photoreceptor of hypothetical converter is unlikely.

The neutral flavin semiquinone, absorbs light of a wide spectral range. Its long-wavelength absorption band has maxima at 502 and 580 nm, and in non-polar solvents its molecules can absorb photons of almost the entire visible spectrum [83]. As for the anionic form of quinone, it absorbs light at about the same shortwave region as the oxidized molecule [84]. The molar absorptivity coefficient of semiquinone, especially its anionic form, is significantly lower than that of oxidized flavins (ɛ_max_ ≤ 5.0 mM^−1^·cm^−1^) [83,84]. The main obstacle to the participation of semiquinone in the primitive energy converter is its instability. In their unbound state in solution, the flavin semiquinone molecules are short-lived [85]. They react by disproportionation, the rate constant (2 k) for which has been measured as 1.2 × l0^9^ M^−1^·s^−1^ for the neutral and 7 × 10^8^ M^−1^·s^−1^ for the anionic riboflavin semiquinone in aqueous solution [86]. Binding to a specific protein dramatically increases the semiquinone stability and some enzymes give almost 100% stabilization of the flavin free radical [16,87]. Association with some non-protein matrices also increase the stability of flavin semiquinone. The role of such a matrix can be, for example, a starch film. In the study of the flavin photocycle in these conditions the appearance of semiquinone was recorded without using time-resolved spectroscopy [88]. However, there is no evidence that the matrices available in the epoch of primitive evolution, for example a mineral surface, could stabilize the semiquinone. In addition, the very fact of the photo-chemical activity of quinone requires clarification. In the absence of conditions providing stable and sufficiently high concentration of the semiquinone, its functioning as a receptor of light becomes unlikely.

In this context, when attempting to outline the conditions that are necessary for the implementation of the flavin photocycle, a prerequisite is the availability of a reoxidizing agent, which does not necessarily need to be atmospheric dioxygen. For example, a suitable candidate for this role is hydrogen peroxide, which ensures the functioning of the flavin-based photophosphorylation in the model [33]. Curiously, low oxygen tension in the atmosphere is not an obstacle for the geochemical formation of hydrogen peroxide: An effective source of hydrogen peroxide can be water photolysis catalyzed by iron-sulfur minerals [89]. A variety of compounds could serve as the electron source for the reduction of an electrophilic molecule of excited flavin in organisms and in the postulated abiotic environment; these compounds include carboxylic acids, alcohols and sulfides [17,18]. In this respect, the flavin model is comparable to early (anoxygenic) versions of photosynthesis that cannot utilize water as an electron source, and the flavin model obtains electrons from the oxidation of sulfides, organic acids and hydrogen.

## 9. Could Flavin Compete with Chlorophyll in the Evolution of Solar Energy Converters?

Why did flavins not occupy the energy converter niche in the biosphere? The answer, apparently, lies in the fact that flavin activity is limited to short-wavelength visible light and the UV-A region. The development of the antenna could only expand this area at the expense of UV photons. Chlorophyll, particularly when supplemented with an antenna, collects energy from the total visible spectrum, and the photon energy in any region of the visible spectrum is sufficient for the formation of energy-rich ATP molecules. For instance, the energy of blue-region photons (λ = 450 nm) is 266 kJ·mol^−1^, and it is equal to 178 kJ·mol^−1^ for the photons of the red area (λ = 670 nm). Furthermore, the energy of the hydrolytic cleavage of the terminal phosphoanhydride bond in ATP is −30.5 kJ·mol^−1^ (under typical cellular conditions, this value is higher, *i.e.*, −57 kJ·mol^−1^) [90]. Moreover, the mechanism of photosynthetic phosphorylation requires two photons to attach one phosphate residue to ADP.

Two points should be taken into account when discussing the selection of molecules for the role of chromophores in photoreceptors. The first point is that flavin molecules have a lower absorptivity than chlorophyll: The molar absorption coefficient for the long-wavelength absorption maximum of chlorophyll a (ɛ_662_) is 91.2 mM^−1^·cm^−1^, whereas the corresponding value for flavin (for example, FMN) at 450 nm is only 12.1 mM^−1^·cm^−1^. The second important point is the ability of photosynthetic pigments (RC plus antenna) to absorb light from nearly the entire visible region. Supplementation of the flavin reaction center with an antenna expands the absorption spectrum to include the ultraviolet region. This is true not only with respect to the oxidized flavin, but also the dihydroform, and the participation of flavin semiquinone is unlikely due to its instability. For these reasons, flavins could not compete for the role of the main converter of light energy in the biosphere.

However, we should not underestimate the importance of flavins and such chemically related compounds as pterins in the evolution of photobiological processes. For the selection of molecules that are suitable to act as photoreceptor chromophores, it is important that these coenzyme molecules are always available in the cell. The presence of additional classes of photoreceptor chromophores, such as chlorophylls, necessitates specific biosynthetic pathways that require significant energy and information costs. In contrast, flavins are essential components of metabolic systems and are always present. As a consequence, evolution did not eliminate flavins from this role. The activity of excited flavins in electron and energy transfer has ensured them an important niche in the light-controlled adaptation and regulatory processes specifically associated with the absorption of blue light and UV-A radiation.

## 10. Conclusions

The ability of flavins to transform light energy to ATP energy, along with information regarding the function of modern flavoprotein photoreceptors, specifically the fact that evolution has repeatedly incorporated flavins into light-reception processes, indicates the possibility of their involvement in the activity of early energy-converting photosystems. These photosystems are homologous to photosynthetic phosphorylation. Analysis of the modern photoreceptor proteins suggests that evolution could have provided them with an antenna that intensifies the flow of excitation energy to the photocatalytic center. Despite the fact that such a converter could be effective, it is hard to imagine that it served as a direct precursor to the development of bioenergetic systems that operate in modern organisms. We are not yet able to trace the continuity between the development of abiogenic chemical models and the evolution of organisms based on the operation of informational macromolecules. In modern organisms, the structure of proteins and other participants of the process from generation to generation are determined by genetic programs. Furthermore, the structure of an abiogenic system is not genetically fixed, and any shift in the parameters of the medium leads to major changes in its structural and functional properties. Therefore, the potentially successful abiogenic prototypes of metabolic devices could not be the direct ancestors of modern bioenergetic mechanisms, the structures of which are genetically determined. Future discussions will involve the physical and chemical aspects of these processes to build hypotheses about the probability of certain versions of the evolutionary process.

## Figures and Tables

**Table 1:** Parameters of existing and hypothetical light energy converters.

Parameter	Photosynthetic apparatus	Bacteriorhodopsin-driven mechanism	Flavin-based system	
			Flavoprotein photoreceptors	Model of abiogenic photophosphorylation
Chromophore of the photochemically active pigment	Mg-porphyrin Chlorophyll or bacteriochlorophyll	Isoprenoid Retinal (*all*-*trans* and 13-*cis*)	Isoalloxazine Flavin (FMN, FAD)	Isoalloxazine, Pteridine
Antenna pigments	Mg-porphyrin (Chlorophylls or bacteriochlorophylls Polyene (carotenoids) Linear tetrapyrrole (bilins)	No	Deazaflavin, Pterin (MTHF), Isoalloxazine (FMN, FAD)	No data available
Active spectral range, nm	approx. 400/800	approx. 500/650	approx. 320/500 (For oxidized molecules)	approx. 320/500 (For oxidized molecules)
Microenvironment of the reaction center (photocatalytic center) and antenna	Lipid membrane	Lipid membrane	Protein molecule in aqueous medium	Matrix surrounded with aqueous medium
Involvement of excited pigment in energy transfer	Yes	No	Yes (in some photoreceptors)	No data available
Conversion of photon energy into the energy of ATP	Yes	Yes	No	Yes
N_AP_/N_RCP_*	≈ 10/10^3^	No	= 1	No data available

* Stoichiometry of the antenna and photocatalytic (the reaction center) pigments.

## References

[1] Schopf J.W. (2006). Fossil evidence of Archaean life. Phil. Trans. R. Soc. B.

[2] Blankenship R.E. (2010). Early evolution of photosynthesis. Plant Physiol.

[3] Whitmarsh J., Govindjee, Singhal G.S., Renger G. (1999). The Photosynthetic Process. Concepts in Photobiology: Photosynthesis and Photomorphogenesis.

[4] Osterhelt D. (1998). The structure and mechanism of the family of retinal proteins from halophilic Archaea. Curr. Opin. Struct. Biol.

[5] Fischer M., Schott A.K., Romisch W., Ramsperger A., Augustin M., Fidler A., Bacher A., Richter G., Huber R., Eisenreich W. (2004). Evolution of vitamin B_2_ biosynthesis. A novel class of riboflavin synthase in Archaea. J. Mol. Biol.

[6] Zylberman V., Klinke S., Haase I., Bacher A., Fisher M., Goldbaum F.A. (2006). Evolution of vitamin B_2_ biosynthesis: 6,7-dimethyl-8-ribityllumazine synthases of *Brucella*. J. Bacteriol.

[7] Heinz B., Ried W., Dose K. (1979). Thermische erzeugung von pteridinen und flavinen aus aminosäueregemischen. Angew. Chem.

[8] Heinz B., Ried W. (1981). The formation of chromophores through amino acid thermolysis and their possible role as prebiotic photoreceptors. BioSystems.

[9] Kolesnikov M.P., Kritsky M.S. (2001). Study of chemical structure and of photochemical activity of abiogenic flavin pigment. J. Evol. Biochem. Physiol.

[10] Fox S.W., Dose K. (1972). Molecular Evolution and the Origin of Life.

[11] Lai E.C. (2003). RNA sensors and riboswitches: Self-regulating messages. Curr. Biol.

[12] Kritsky M.S., Telegina T.A., Seckbach J. (2004). Role of Nucleotide-Like Coenzymes in Primitive Evolution. Role of Nucleotide-Like Coenzymes in Primitive Evolution. Origins: Genesis, Evolution and Diversity of Life.

[13] Heelis P.F. (1982). The photophysical and photochemical properties of flavins (izoalloxazines). Chem. Soc. Rev.

[14] Massey V. (2000). The chemical and biological versatility of riboflavin. Biochem. Soc. Trans.

[15] Hemmerich P., Schmidt W., Lenci F., Colombetti G. (1979). Bluelight Reception and Flavin Photochemistry. Photoreception and Sensory Transduction in Aneural Organisms.

[16] Knappe W.R., Hemmerich P. (1972). Covalent intermediates in flavin-sensitized photodehydrogenation and photodecarboxylation. Z. Naturforsrh. Teil B.

[17] Vincent V., Stankovich M., Hemmerich P. (1978). Light-mediated reduction of flavoproteins with flavins as catalysts. Biochemistry.

[18] Holmstrom B. (1964). Flash photoreduction of flavin mononucleotide in neutral solutions. Absolute absorptivity of the semiquinone. Photochem. Photobiol.

[19] Vaish S.P., Tollin G. (1970). Flash photolysis of flavins. IV. Some properties of the lumiflavin triplet state. Bioenergetics.

[20] Vaish S.P., Tollin G. (1971). Flash photolysis of flavins. V. Oxidation and disproportionation of flavin radicals. J. Bioenerg.

[21] Brüstlein M., Knappe W.R., Hemmerich P. (1971). Novel photoalkylation reactions on the flavin nucleus. Angew. Chem. Int. Ed. Engl.

[22] Hemmerich P., Haas W., Yagi K. (1975). Recent Developments in the Study of “Fully Reduced Flavin”. Reactivity of Flavins.

[23] Heelis P.F., Hartman R.F., Rose S.D. (1995). Photoenzymic repair of UV-damaged DNA: A chemist’s perspective. Chem. Soc. Rev.

[24] Egorov S.Y., Krasnovsky A.A., Bashtanov M.Y., Mironov E.A., Ludnikova T.A., Kritsky M.S. (1999). Photosensitization of singlet oxygen formation by pterins and flavins. Time-resolved studies of oxygen phosphorescence under laser excitation. Biochemistry-Moscow.

[25] Baier J., Maisch T., Maier M., Engel E., Landthaler M., Bäumler W. (2006). Singlet oxygen generation by UVA light exposure of endogenous photosensitizers. Biophys. J.

[26] Krasnovskii A.A., Chernysheva E.K., Kritskii M.S. (1987). Investigation of the role of active forms of oxygen in flavin-photosensitized oxidation of NADH. Biochemistry-Moscow.

[27] Acworth I.N. (2003). The Handbook of Redox Biochemistry.

[28] Frisell W.R., Chung C.W., Mackenzie C.G. (1959). Catalysis of oxidation of nitrogen compounds by flavin coenzymes in the presence of light. J. Biol. Chem.

[29] Vernon L.P. (1959). Photochemical oxidation and reduction reactions catalyzed by flavin nucleotides. Biochim. Biophys. Acta.

[30] Song S.-H., Dick B., Penzkofer A. (2007). Photo-induced reduction of flavin mononucleotide in aqueous solutions. Chem. Phys.

[31] Bladel W.T., Laessig R.H. (1965). Continous EDTA titrations with a dropping mercury electrode. Automated titrations based on nonsymmetric curves. Anal. Chem.

[32] Schmidt W., Butler W.L. (1976). Flavin-mediated photoreactions in artificial systems—Possible model for blue-light photoreceptor pigment in living systems. Photochem.

[33] Kolesnikov M.P., Telegina T.A., Lyudnikova T.A., Kritsky M.S. (2008). Abiogenic photophosphoryation of ADP to ATP sensitized by flavoproteinoid microspheres. Orig. Life Evol. Biosph.

[34] Kolesnikov M.P. (1991). Proteinoid microspheres and the process of prebiological photophosphorylation. Orig. Life Evol. Biosph.

[35] Kritsky M.S., Kolesnikov M.P., Telegina T.A. (2007). Modeling of abiogenic synthesis of ATP. Doklady Biochem. Biophys.

[36] Svoboda J., König B. (2006). Templated photochemistry: Toward catalysts enhancing the efficiency and selectivity of photoreactions in homogeneous solutions. Chem. Rev.

[37] Telegina T.A., Kolesnikov M.P., Vechtomova Y.L., Kritsky M.S. (2012). Abiotic photophosphorylation model based on abiogenic flavin and pteridine pigments.

[38] Losinova T.A., Nedelina O.S., Kayushin L.P. (1986). Effect of adenosine diphosphate on the light-dependent oxygen absorption by flavins. Biophysics.

[39] Nedelina O.S. (1997). Biosynthesis of ATP. Elementary Chemical Act of ATP Synthesis in Oxidative Phosphorylation.

[40] Maheen G., Tian G., Wang Y., He C., Shi Z., Yuan H., Feng S. (2011). Mimicking the prebiotic acidic hydrothermal environment: One-pot prebiotic hydrothermal synthesis of glucose phosphates. Heteroat. Chem.

[41] Krasnovskii A.A., Oparin A.I., Pasynskii A.G., Braunstein A.E., Pavlovskaya T.E., Clark F., Synge R.L.M. (1959). Development of the Mode of Action of the Photocatalytic System in Organisms.

[42] Sancar A. (2003). Structure and function of DNA photolyase and cryptochrome blue-light photoreceptors. Chem. Rev.

[43] Sancar A. (2008). Structure and function of photolyase and *in vivo* enzymology: 50th anniversary (minireview). J. Biol. Chem.

[44] Partch C.L., Sancar A. (2005). Photochemistry and photobiology of cryptochrome blue-light photopigments: The search for a photocycle. Photochem. Photobiol.

[45] Müller M., Carell T. (2009). Structural biology of DNA photolyases and cryptochromes. Curr. Opin. Struct. Biol.

[46] Losi A. (2007). Flavin-based blue-light photosensors: A photobiophysics update. Photochem. Photobiol.

[47] Losi A., Gaertner W. (2011). Old chromophores, new photoactivation paradigms, trendy applications: Flavins in blue light-sensing photoreceptors. Photochem. Photobiol.

[48] Metz S., Jaeger A., Klug G. (2009). *In vivo* sensitivity of blue-light-dependent signaling mediated by AppA/PpsR or PrrB/PrrA in *Rhodobacter sphaeroides*. J. Bacteriol.

[49] Schafmeier T., Diernfellner A.C.R. (2011). Light input and processing in the circadian clock of *Neurospora*. FEBS Lett.

[50] Swartz T.E., Tseng T.-S., Frederickson M.A., Paris G., Comerci D.J., Rajashekara G., Kim J.-G., Mudgett M.B., Splitter G.A., Ugalde R.A.G. (2007). Blue-light-activated histidine kinases: Two-component sensors in bacteria. Science.

[51] Barends T.R.M., Hartmann E., Griese J.J., Beitlich T., Kirienko N., Ryjenkov D., Reinstein J., Shoeman R., Gomelsky M., Schlichting I. (2009). Structure and mechanism of a bacterial light-regulated cyclic nucleotide phosphodiesterase. Nature.

[52] Ito S., Murakami A., Iseki M., Takahashi T., Higashi S., Watanabe M. (2010). Differentiation of photocycle characteristics of flavin-binding BLUF domains of α-β-subunits of photoactivated adenylyl cyclase of *Euglena gracilis*. Photochem. Photobiol. Sci..

[53] Kritsky M.S., Telegina T.A., Vechtomova Y.L., Kolesnikov M.P., Lyudnikova T.A., Golub O.A. (2010). Excited flavin and pterin coenzyme molecules in evolution. Biochemistry.

[54] Corrochano L.M. (2007). Fungal photoreceptors: Sensory molecules for fungal development and behaviour. Photochem. Photobiol. Sci.

[55] Gauden M., Stokkum I.H.M., van Ihalainen J.A., Grondelle R., van Kennis J.T.M., Yeremenko S., Laan W., Hellingwerf K.J. (2005). Photocycle of the flavin-binding photoreceptor APPA, a bacterial transcriptional antirepressor of photosynthesis genes. Biochemistry.

[56] Dragnea V., Waegle M., Balascuta S., Bauer C., Dragnea B. (2005). Time-resolved spectroscopic studies of the AppA blue-light receptor BLUF domain from *Rhodobacter sphaeroides*. Biochemistry.

[57] Salomon M., Christie J.M., Knieb E., Lempert U., Briggs W.R. (2000). Photochemical and mutational analysis of the FMN-binding domains of the plant blue light receptor, phototropin. Biochemistry.

[58] Stokkum I.H.M., van Gauden M., Crosson S., Grondelle R., van Moffat K., Kennis J.T.M. (2011). The primary photophysics of the *Avena sativa* phototropin 1 LOV2 domain observed with time-resolved emission spectroscopy. Photochem. Photobiol.

[59] Brudler R., Hitomi K., Diayasu H., Toh H., Kucho K., Ishiura M., Kanehisa M., Roberts V.A., Todo T., Tainer J.A. (2003). Identification of a new cryptochrome class. Structure, function, and evolution. Mol. Cell.

[60] Swartz T.E., Corchnoy S.B., Christie J.M., Lewis J.L., Schundi W.R., Briggs W.R., Bogomolni R.A. (2001). The photocycle of flavin binding domain of the blue light photoreceptor phototropin. J. Biol. Chem.

[61] Dittrich M., Freddolino P.L., Schulten K. (2005). When light falls in LOV: A quantum mechanical/molecular mechanics study of photoexcitation in Phot-LOV1 of *Chlamydomonas reinhardtii*. J. Phys. Chem. B.

[62] Gauden M., Stokkum I.H.M., van Key J.M., Luhrs D.C., Grondelle R., van Hegemann P., Kennis J.T.M. (2006). Hydrogen-bond switching through a radical pair mechanism in a flavin-binding photoreceptor. Proc. Natl. Acad. Sci. USA.

[63] Bonetti C., Mathes T., Stokkum I.H.M., van Mullen K.M., Groot M.-L., Grondelle R., van Hegemann P., Kennis J.T.M. (2008). Hydrogen bond switching among flavin and amino acid side chains in the BLUF photoreceptor observed by ultrafast infrared spectroscopy. Biophys. J.

[64] Bonetti C., Stierl M., Mathes T., Stokkum I.H.M., van Mullen K.M., Cohen-Stuart T.A., Grondelle R., Hegemann P., van Kennis J.T.M. (2009). The role of key amino acids in the photoactivation pathway of the *Synechocystis Slr1694* BLUF domain. Biochemistry.

[65] Mathes T., Stokkum I.H.M., van Bonetti C., Hegemann P., Kennis J.T.M. (2011). The hydrogen-bond switch reaction of the Blrb Bluf Domain of *Rhodobacter sphaeroides*. J. Phys. Chem. B.

[66] Green B.R., Green B.R., Parson W.W. (2003). The Evolution of Light-Harvesting Antennas. Light-Harvesting Antennas in Photosynthesis (Advances in Photosynthesis and Respiration).

[67] Jordan P., Fromme P., Witt H.T., Klukas O., Saenger W., Krauβ N. (2001). Three-dimensional structure of cyanobacterial photosystem I at 2.5Å resolution. Nature.

[68] Dawson M.C., Elliott D.C., Elliott W.H., Jones K.M. (1986). Data for Biochemical Research.

[69] Brown S.B., Holroyd J.A., Troxler R.F., Offenert G.D. (1981). Bile pigment synthesis in plants. Incorporation of haem into phycocyanobilin and phycobiliproteins in *Cyanidium caldarium*. Biochem. J.

[70] Zechmeister L. (1962). *cis-trans* Isomeric Carotenoids Vitamins A and Arylpolyenes.

[71] Lucock M.D. (2000). Folic acid: Nutritional biochemistry, molecular biology, and role in disease processes (minireview). Mol. Genet. MeTable.

[72] Eirich L.D., Vogels G.D., Wolfe R.S. (1978). Proposed structure for coenzyme F420 from *Methanobacterium*. Biochemistry.

[73] Rabinowitz J.C. (1963). Preparation and properties of 5,10-methenyltetrahydrofolic acid and 5-formyltetrahydrofolic acid. Methods Enzymol.

[74] Bair T.B., Isabelle D.W., Daniels L. (2001). Structures of coenzyme F420 in *Mycobacterium* species. Arch. Microbiol.

[75] Klar T., Kaiser G., Hennecke U., Carell T., Batschauer A., Essen L-O. (2006). Natural and non-natural antenna chromophores in the DNA photolyase from *Thermus thermophilus*. Chem Bio Chem.

[76] Fujihashi M., Numoto N., Kobayashi Y., Mizushima A., Tsujimura M., Nakamura A., Kawarabayasi Y., Miki K. (2007). Crystal structure of archaeal photolyase from *Sulfolobus tokodaii* with two FAD molecules: Implication of a novel light-harvesting cofactor. J. Mol. Biol.

[77] Geisselbrecht Y., Frühwirth S., Schroeder C., Pierik A.J., Klug G., Essen L.-O. (2012). CryB from *Rhodobacter sphaeroides*: A unique class of cryptochromes with new cofactors. EMBO Reports.

[78] Telegina T.A., Lyudnikova T.A., Zemskova Y.L., Kritsky M.S. (2005). Resistance of 5,10-methenyltetrahydrofolate to ultraviolet radiation. Appl. Biochem. Microbiol.

[79] Schmidt W. (1984). Bluelight-induced, flavin-mediated transport of redox equivalents across artificial bilayer membranes. J. Membr. Biol.

[80] Kaler J.B. (1997). Stars and Their Spectra: An Introduction to the Spectral Sequence.

[81] Ahmed T., Elert G. (2002). The Wavelength of the Sun’s Peak Radiation Output. The Physics Factbook—An Encyclopedia of Scientific Essays.

[82] Holland H.D. (2006). The oxygenation of the atmosphere and oceans. Phil. Trans. Royal Soc.

[83] Müller F., Brüstlein M., Hemmerich P., Massey V., Walker W.H. (1972). Light-absorption studies on neutral flavin radicals. Eur. J. Biochem.

[84] Ehrenberg A., Müller F., Hemmerich P. (1967). Basicity, visible spectra, and electron spin resonance of flavosemiquinone anions. Eur. J. Biochem.

[85] Su Y., Tripathi G.N.R. (1994). Time-resolved resonance Raman observation of protein-free riboflavin semiquinone radicals. J. Am. Chem. Soc.

[86] Land E.J., Swallow A.J. (1969). One-electron reactions in biochemical systems as studied by pulse radiolysis. II. Riboflavin. Biochemistry.

[87] Edwards A.M., Silva E., Edwards A.M. (2006). General Properties of Flavins. Flavins: Photochemistry and Photobiology. Comprehensive Series in Photochemistry and Photobiology.

[88] Penzkofer A. (2012). Reduction-oxidation photocycle dynamics of flavins in starch films. Int. J. Mol. Sci.

[89] Borda M.J., Elsetinow A.R., Schoonen M.A., Strongin D.R. (2001). Pyrite-induced hydrogen peroxide formation as a driving force in the evolution of photosynthetic organisms on an early Earth. Astrobiology.

[90] Berg J.M., Tymoczko J.L., Stryer L. (2007). Biochemistry.

